# Divergent and point-to-point connections in the commissural pathway between the inferior colliculi

**DOI:** 10.1002/cne.21997

**Published:** 2009-05-20

**Authors:** Manuel S Malmierca, Olga Hernández, Flora M Antunes, Adrian Rees

**Affiliations:** 1 Auditory Neurophysiology Unit, Institute for Neuroscience of Castilla y León37007 Salamanca, Spain; 2 Department Cell Biology and Pathology, Faculty of Medicine, University of Salamanca, and Institute for Neuroscience of Castilla y León37007 Salamanca, Spain; 3 Auditory Group, Institute of Neuroscience, Faculty of Medical Sciences, Newcastle University, Newcastle upon TyneNE2 4HH United Kingdom

**Keywords:** auditory system, tract tracing, computer-assisted 3D reconstructions, cytoarchitecture, tonotopic organization

## Abstract

The commissure of the inferior colliculus interconnects the left and right sides of the auditory midbrain and provides the final opportunity for interaction between the two sides of the auditory pathway at the subcortical level. Although the functional properties of the commissure are beginning to be revealed, the topographical organization of its connections is unknown. A combination of neuroanatomical tracing studies, 3D reconstruction, and neuronal density maps was used to study the commissural connections in rat. The results demonstrate that commissural neurons in the central nucleus of the inferior colliculus send a divergent projection to the equivalent frequency-band laminae in the central nucleus and dorsal and lateral cortices on the opposite side. The density of this projection, however, is weighted toward a point that matches the position of the tracer injection; consistent with a point-to-point emphasis in the wiring pattern. In the dorsal cortex of the inferior colliculus there may be two populations of neurons that project across the commissure, one projecting exclusively to the frequency-band laminae in the central nucleus and the other projecting diffusely to the dorsal cortex. Neurons in the lateral cortex of the inferior colliculus make only a very weak contribution to the commissural pathway. The point-to-point pattern of connections permits interactions between specific regions of corresponding frequency-band laminae, whereas the divergent projection pattern could subserve integration across the lamina. J. Comp. Neurol. 514:226–239, 2009. © 2009 Wiley-Liss, Inc.

The commissure of the inferior colliculus (CoIC) is a prominent fiber tract interconnecting the two inferior colliculi and provides the last opportunity for interaction between the left and right sides of the auditory pathway at the subcortical level (Adams,^1979^,^1980^; Aitkin and Phillips,^1984^; Coleman and Clerici,^1987^; Gonzalez-Hernandez et al.,^1986^,^1996^). The functional significance of the commissural projection is not fully specified, but we have demonstrated that it modulates the responses of IC neurons to sounds (Malmierca et al.,^2003^,2005a).

A wealth of studies has described how ascending projections from the brainstem and descending projections from the auditory cortex terminate in the three main subdivisions of the IC (Adams,^1979^; Andersen et al.,^1980^; Brunso-Bechtold et al.,^1981^; Cant and Benson,^2003^,^2006^; Malmierca et al.,^1998^,^2002^,2005b; Oliver,^1987^; Oliver and Shneiderman,^1989^; Oliver et al.,^1995^,^1997^; Roth et al.,^1978^; Saldana et al.,^1996^; Schofield and Cant,^1996^; Whitley and Henkel,^1984^; Winer et al.,^1998^). In contrast, the organization of the commissural connections with respect to these subdivisions has received little attention. Most studies of the commissural projection, dating from the 1980s, employed large injections of horseradish peroxidase (HRP; Adams,^1980^; Aitkin and Phillips,^1984^; Beyerl,^1978^; Brunso-Bechtold et al.,^1981^; Coleman and Clerici,^1987^; Gonzalez-Hernandez et al.,^1986^) and so could not resolve the details of connections between subdivisions.

Studies using anterograde tracers demonstrated connections between the frequency-band laminae in one IC and the equivalent laminae in the contralateral IC (Malmierca et al.,1995a; Saldaña and Merchán,^1992^). Malmierca et al. (1995a) proposed two possible schemes of organization that could account for the pattern of labeling observed: a point-to-point pattern of connections in which specific points on a lamina connect equivalent points on the other side, or a divergent pattern in which a single point on the lamina provides widespread connections to the whole of the contralateral lamina. Their data, however, could not differentiate between these two models.

In this study, we combine tract tracing using two different tracers injected at known frequency locations with computer-assisted 3D reconstructions to test the hypotheses that 1) distinct patterns of commissural connections occur between different subdivisions of the IC and 2) a point-to-point pattern of connections exists between frequency-band laminae.

The distribution of commissural neurons labeled by a single small injection in a frequency-band lamina is consistent with the notion that a point on the lamina makes divergent connections throughout the lamina in the opposite IC but that the density of connections is greatest between corresponding points. Although the projections of some commissural neurons in the dorsal cortex connect to the contralateral dorsal cortex, another population projects only to the central nucleus, connecting these two subdivisions. We conclude that the connections of the CoIC mediate integration both within frequency-band laminae and across different subdivisions of the IC.

## MATERIALS AND METHODS

### Animal preparation, surgery, and tracer injections

Experiments were performed on adult pigmented rats (*Rattus norvergicus*, Rj: Long Evans) of either sex weighing between 180 and 420 g (n = 10). All procedures were approved by and conformed to the standards of the University of Salamanca Animal Care Committee.

An areflexive anesthetic state was induced via an intramuscular injection of a mixture of ketamine hydrochloride (60 mg · kg^−1^) and xylazine (Rompun; 16 mg · kg^−1^) and supplemented with the same mixture to maintain suppression of the pedal withdrawal reflex. Atropine sulfate (600 μl · ml^−1^; Phoenix Pharma) was administered subcutaneously to suppress bronchial secretions. Body temperature was maintained at 38°C with a thermostatically controlled blanket. The animal was placed in a stereotaxic frame in which the ear bars were replaced by hollow speculi that accommodated a sound delivery system. A craniotomy was performed to expose the cerebral cortex overlying the inferior colliculi on one side, and the animal was situated inside a double-walled sound-attenuating enclosure.

Tracer injections were made with glass micropipettes (tip diameter 10–40 μm) filled with either 10% tetramethyl-rhodamine-dextran (TRD) dissolved in 0.9% saline or a mixture of 10% fluorescein-dextran (FD) and 10% biotinylated dextran amine (BDA) in 0.9% saline (Malmierca et al.,^2002^,2005b; Oliver et al.,^1997^). Pipettes were advanced into the IC with a microdrive (Burleigh Instruments Inc., Victor, NY), and, once the desired positions were located, the dextrans were injected by iontophoresis (2–6 μA for 5–24 minutes). The best frequency (sound frequency requiring the least intensity to drive the neuron) at each site of tracer injection was identified by using the pipettes to make extracellular recordings of neuronal activity in response to acoustic stimulation. Pure tones presented at the neuron's best frequency (BF) or noise bursts were generated under computer control and delivered through a closed acoustic system (Malmierca et al.,^2003^,2005a; Rees et al.,^1997^). Stimuli were 75 msec in duration with 5-msec rise/fall times. Action potentials were amplified (×10,000), filtered (0.3–3 kHz), and window discriminated. Best frequency was defined audiovisually.

**Figure 1:**
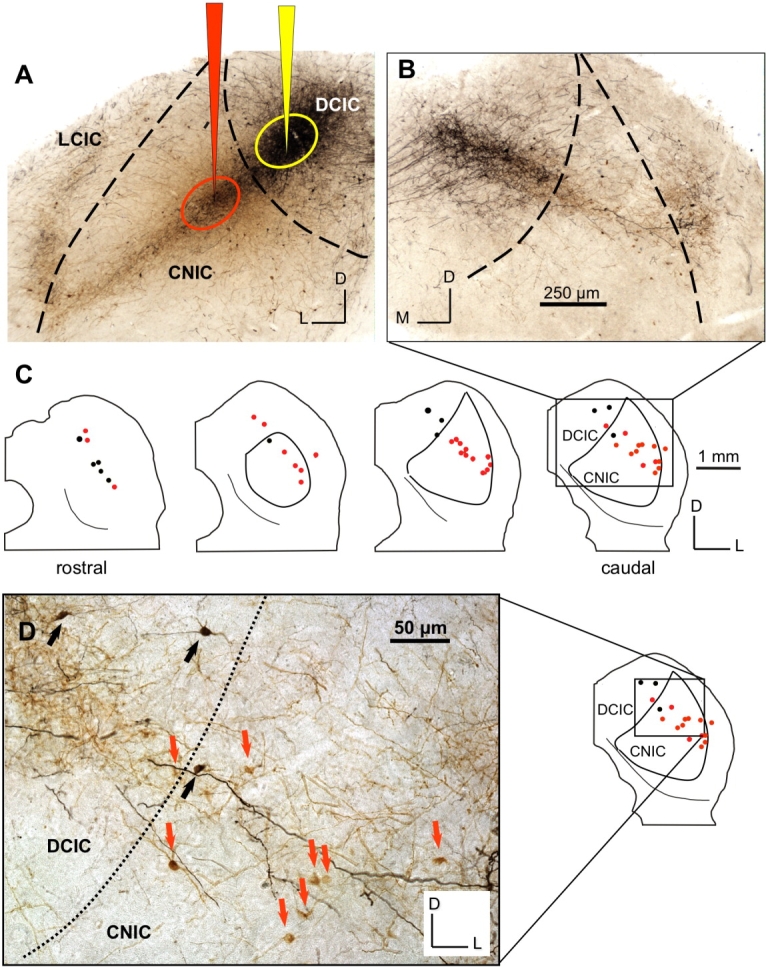
**A**: Photomicrograph showing two injections into the same lamina of the IC (case 279). The FD-BDA injection is confined to the DCIC, whereas the TRD injection is located in the CNIC. The BF at the injection sites was 10–10.5 kHz. Note the typical V-shaped plexus of intrinsic axons with a central wing located in the CNIC that extends into the DCIC and a lateral wing in the lateral cortex. The vertex of the plexus marks the border between the CNIC and the LCIC. **B**: Anterogradely labeled axons and retrogradely labeled neurons on the side contralateral to the injections in A. **C**: Camera lucida drawings of the retrogradely labeled neurons originating from the TDR and FD-BDA injections marked in red and black, respectively, at different rostrocaudal levels of the IC. Boxed area in the most caudal section is the area shown in B. **D**: High-magnification photograph of boxed area in the caudalmost section in C. Red arrows point to TRD-labeled neurons and black arrows to FD-BDA-labeled neurons. Scale bars = 250 μm in B (applies to A,B); 50 μm in D. [Color figure can be viewed in the online issue, which is available at http://www.interscience.wiley.com.]

Seven to ten days after the injections, the brains were perfusion fixed and prepared for light microscopy. Under deep surgical anesthesia, the animals were perfused transcardially with a buffered washout solution (2% sucrose in 0.12 M phosphate buffer, pH 7.4, containing 0.05% lidocaine with 0.004% CaCl_2_), followed by a 4% paraformaldehyde fixative solution. After fixation, decapitation, and dissection to remove the brain, it was cryoprotected in 30% sucrose and sectioned in the transverse plane at 35 μm or 50 μm on a freezing microtome. Single sections underwent avidin-biotin complex histochemistry for FD-BDA (black reaction, Fig. 1), followed by immunohistochemistry with antisera to rhodamine, biotinylated secondary antisera, and avidin-biotin histochemistry (brown reaction, Fig. 1). Standard DAB solution was used for the visualization of TRD, whereas a nickel-enhanced DAB reaction was used to stain neurons showing FD-BDA; therefore every section was labeled for both tracers. Every third or fourth section was counterstained with 0.1% thionin blue to facilitate identification of cytoarchitectural boundaries (Loftus et al.,^2008^; Malmierca et al.,^1993^,1995b). For illustrations, color digital images were captured with a Spot Insight Color CCD camera (Diagnostic Instruments, Sterling Heights, MI), and the contrast and brightness were adjusted in Adobe Photoshop (Adobe, San Jose, CA).

### 3D reconstruction and data analysis

Labeled cells were reconstructed with a Silicon Graphics Indigo SG02 work station (SGI, Mountain View, CA) as described in detail elsewhere (Leergaard et al.,^1995^,^2000^; Malmierca et al.,1995a,^1998^). Briefly, for every section, the positions of *all* the labeled neuronal somata were marked on detailed camera lucida drawings of the IC with a Leitz (Wetzlar, Germany) Diaplan microscope at a total magnification of ×52. Special care was taken to ensure that neurons at the section interface were drawn only once. Six cases were reconstructed in three dimensions (cases 35, 74, 78, 187, 172, 279; see Table 1). The camera lucida drawings were aligned with the aid of the tissue landmarks and fiducial marks and digitized with a modified version of the BioTrace program (Bjaalie and Leergaard,^2006^; Bjaalie et al.,^2006^; Leergaard and Bjaalie,^1995^; Leergaard et al.,^1995^).

**TABLE 1:** Summary of Injections

	TRD		FD-BDA	
Case	Location	BF (kHz)	Location	BF (kHz)
74^1^	CNIC	30	LCIC	30
279^1^	CNIC	10.5	DCIC	10.5
78^1^	LCIC	2	RCIC	2
35^1^	DCIC	2	DCIC	2.7–3
244	CNIC	14.5	CNIC	14.5
110	CNIC	15.5	CNIC	32
187	CNIC	18	CNIC	31
172	LCIC	37	CNIC	36.5
218	RCIC	12–15	BIC	6
105	CNIC	15		

1 Illustrated cases.

To visualize the structures in 3D, the digitized sections were loaded into software developed at the Department of Anatomy, University of Oslo, running on a Silicon Graphics Indigo SGO2 Workstation (Bjaalie and Leergaard,^2006^; Bjaalie et al.,^2006^; Leergaard et al.,^1995^). Tissue shrinkage attributable to the histological processing was estimated to be 10%, and, to maintain correct proportions in the reconstructions, the distances between sections (z-axis) were reduced accordingly. The borders and contours of the IC surrounding the labeling were used to synthesize the surface of the IC. Labeled neurons were recorded as points. The numbers of labeled cells in different parts of the IC were counted automatically by the software. Real-time rotations on the computer screen and stereo images were used to inspect the structures from various angles and to view the objects in depth.

**Figure 2:**
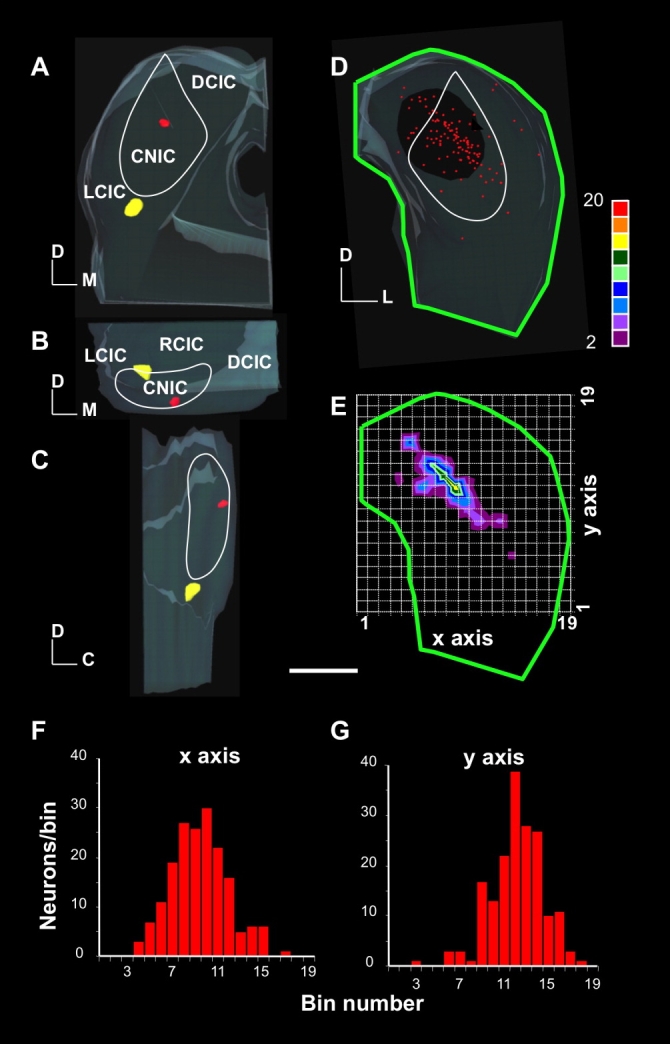
3D reconstruction of the injection sites for two injections in the same animal (case 74) seen from the back (**A**), top (**B**), and lateral (**C**) sides of the IC. **D**: 3D reconstruction of the locations of *all* retrogradely labeled cells originating from the injections. **E**: Grid of ∼150-μm^2^ squares used to produce the neuronal density map of the TRD retrogradely labeled neurons overlain with the grid. **F**,**G**: Histograms of the neuronal distribution across the x and y axes, respectively, of the grid used to generate the density map (cf. E). Note that only red neurons are labeled in the contralateral IC (D) and that the histograms (F,G) show a distinct peak in the number of labeled neurons in both the x and the y axes. CNIC borders, here and elsewhere, are denoted by the solid white line and are based on the cytoarchitectural scheme of the rat (Faye-Lund and Osen,^1985^; Loftus et al.,^2008^; Malmierca et al.,^1993^). Scale bar = 1 mm. [Color figure can be viewed in the online issue, which is available at http://www.interscience.wiley.com.]

**Figure 3:**
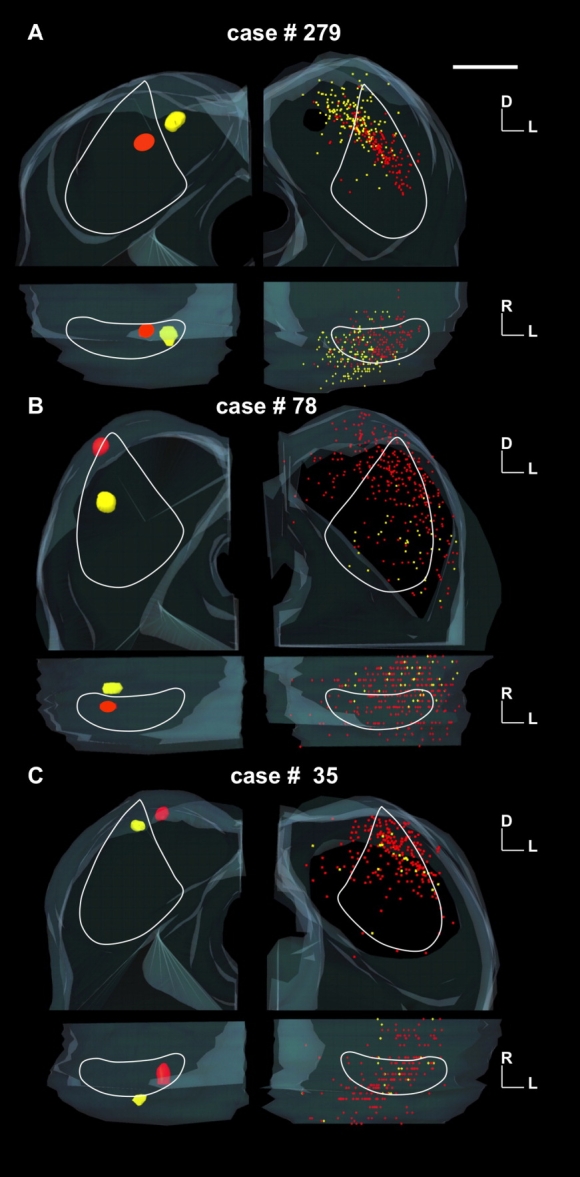
3D reconstructions of the injection sites (left column) and the resulting labeled neurons (right column) in the contralateral ICs derived from injections in three cases. Top parts of each panel show transverse views; bottom parts of each panel show horizontal views. **A**: Case 279 (see also Fig. 4). **B**: Case 78 (see also Fig. 5). **C**: Case 35 (see also Fig. 6). Scale bar = 1 mm. [Color figure can be viewed in the online issue, which is available at http://www.interscience.wiley.com.]

**Figure 4:**
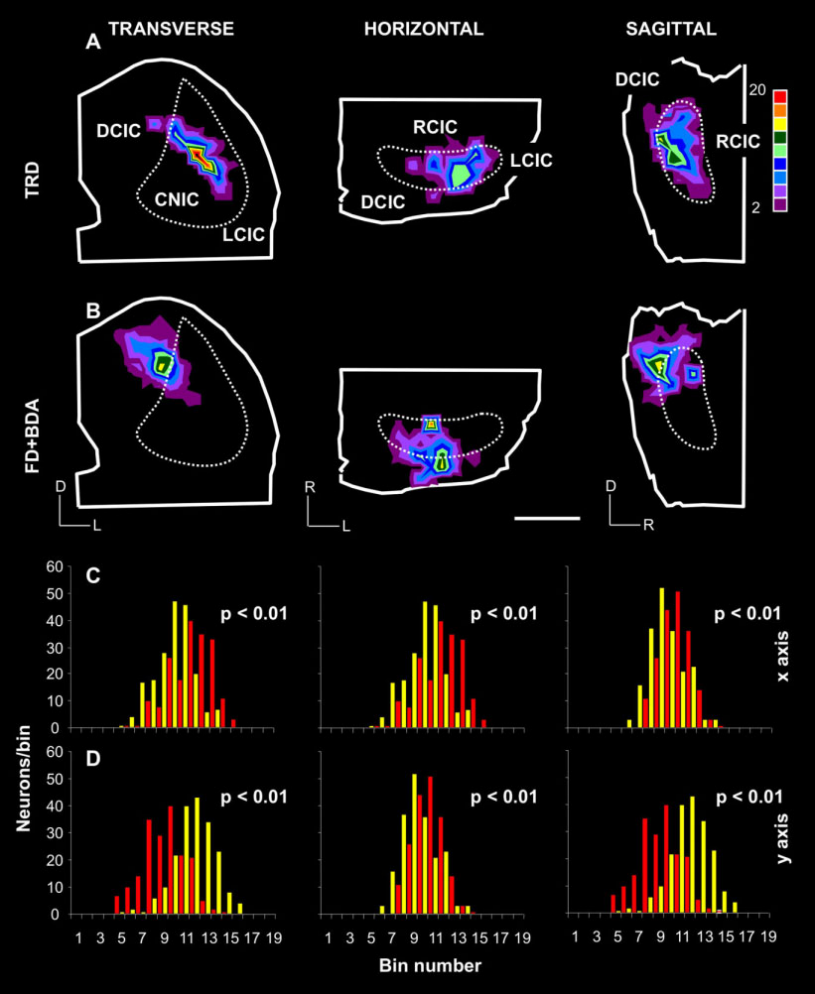
Case 279. **A**: Density maps for TRD-labeled neurons in the transverse, horizontal, and sagittal projections. **B**: Similar projections for the FD-BDA-labeled neurons, following the injections shown in Figure 3A. **C**,**D**: Distribution histograms of the TRD- and FD-BDA-labeled neurons along the x and y axes of the grid (cf. Fig. 2, for clarity not shown). All histograms show an approximately normal distribution, with a distinct peak of neurons. In all projections, the distributions of neurons labeled by the two injections are significantly different. This is particularly evident along the y axis of the transverse and sagittal projections. *P* values show the outcome of *t*-tests comparing the distributions in each histogram. Scale bar = 1 mm. [Color figure can be viewed in the online issue, which is available at http://www.interscience.wiley.com.]

**Figure 5:**
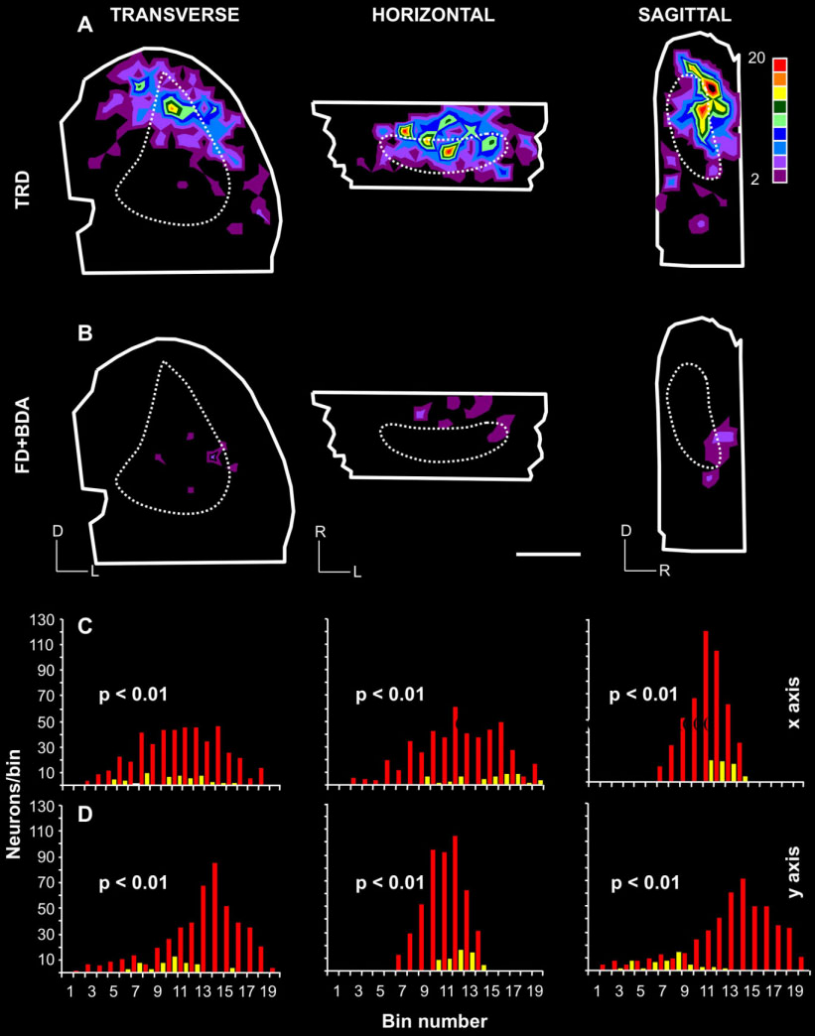
Case 78. Density maps for TRD- and FD-BDA-labeled neurons in the transverse, horizontal, and sagittal projections following the injections shown in Figure 3B. Details for **A**–**D** as for Figure 4. Scale bar = 1 mm. [Color figure can be viewed in the online issue, which is available at http://www.interscience.wiley.com.]

**Figure 6:**
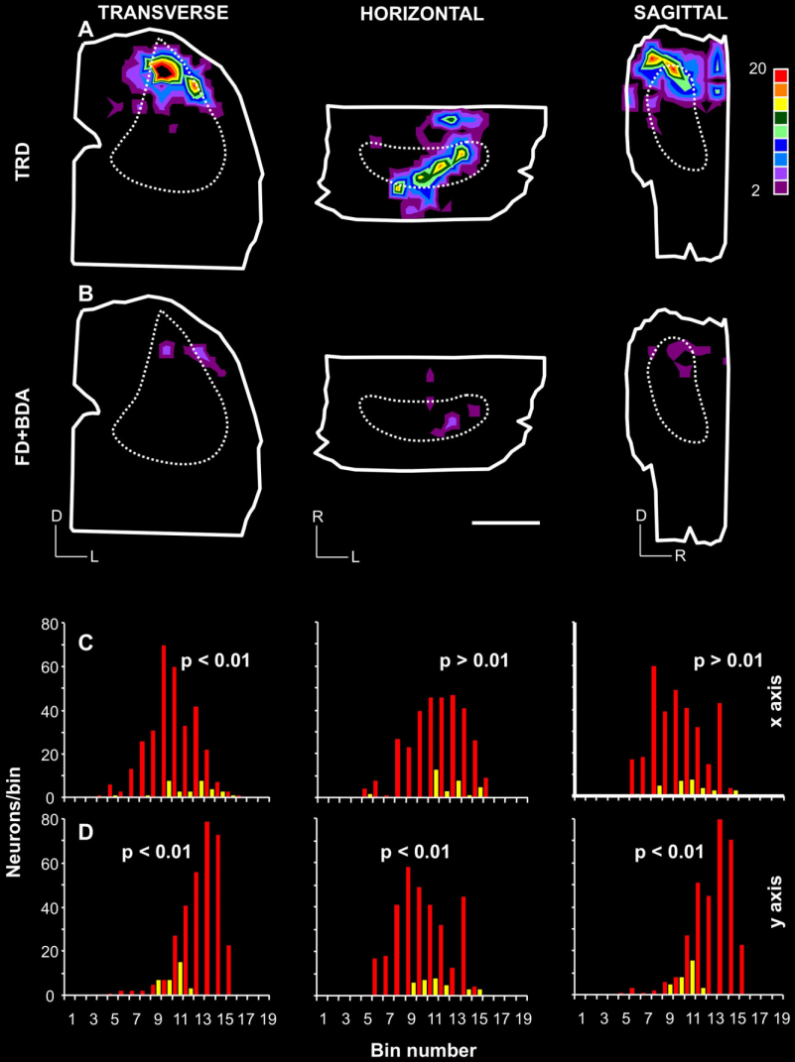
Case 35. A,B: Density maps for TRD- and FD-BDA-labeled neurons in the transverse, horizontal, and sagittal projections following injections shown in Figure 3C. Details for **A**–**D** as for Figure 4. Note the overlap in the distributions of the two populations of labeled neurons. Scale bar = 1 mm. [Color figure can be viewed in the online issue, which is available at http://www.interscience.wiley.com.]

To demonstrate the distribution of labeled neurons, the points representing their positions were used to generate pseudocolor-coded density maps (see Figs. 2–6; cf. Malmierca et al.,^1998^). The density maps were produced by dividing a particular projection (transverse, lateral, or horizontal) of the reconstruction into a grid of ∼150 × 150 μm^2^ (Fig. 2E). Each square was assigned a color representing the number of cells contained within it. For clarity, only squares containing two or more neurons are shown. Histograms of neuronal distribution were generated along the X and Y axes of the grid, and a *t*-test was used to test for differences in the distributions given by the two tracers (see Figs. 2,4–6).

## RESULTS

### Injection sites and general features of labeling

The results presented in this account are based on small injections of two different tracers (TRD and FD-BDA). In six rats (Table 1), a pair of injections was placed at sites tuned to nearly identical frequencies as determined by extracellular multiunit recordings (cases 35, 74, 78, 172, 244, and 279). Two cases had injections at points corresponding to different frequencies (cases 187 and 110), and two cases (cases 105 and 218) had single injections in the IC. A second injection in case 218 was in the brachium of the IC (BIC; Table 1). The center of each injection site could be readily identified (Fig. 1), and each injection of a pair was clearly segregated from the other (Figs. 1A, 2A, 3A–C). After injections in a single frequency-band lamina in one IC (Fig. 1A), the labeling in the contralateral IC (Fig. 1B) includes retrogradely labeled neurons, thick and thin axons, and terminal plexuses, including terminal boutons. The labeled axons arising from the injection site form a terminal plexus that spans the entire CNIC, from ventrolateral to dorsomedial and from caudal to rostral (not shown). The plexus is V-shaped, with the vertex at the border between the CNIC and the lateral cortex. Thus, the plexus defines two wings; a central wing that occupies the CNIC and extends into the DCIC and a lateral wing that extends into the lateral cortex. The location and orientation of the central wing are in register with the fibrodendritic lamina in the CNIC. Neurons labeled with the different tracers were widely distributed in the plane of the axonal plexus, as seen in a series of sections running rostral to caudal (Fig. 1C,D). Detailed analysis of the retrogradely labeled neurons and axonal plexi on the side of the injection that contribute to local connections within the IC (Fig. 1A) is outside of the scope of this study, and these are not considered further. In the following, we focus our attention on the distribution of the retrogradely labeled neurons in the IC contralateral to the injected IC.

### 3D reconstruction of retrograde labeling in the contralateral IC

To discover the relationship between the patterns of labeling that originate from injections into different parts of the same lamina, we made 3D reconstructions of the injection sites in one IC and of the labeled neurons on the contralateral side (see Figs. 2, 3). Our sample includes 10 cases, but in the following paragraphs we describe six cases that have been reconstructed in 3D and are representative of the labeling in all of the cases.

Case 74 (Fig. 2) had a TRD injection in the CNIC and an FD-BDA injection in the ventrolateral corner of the IC. The three different projections of the reconstructed IC showing the injection sites (Fig. 2A–C) demonstrate that the FD-BDA injection is confined to the deep region of the LCIC (Loftus et al.,^2008^). The best frequency recorded at both injection sites was 30 kHz. The distribution of anterograde labeling of axons in the injected IC (not shown) demonstrates that both injections were placed in the same lamina. In the contralateral IC, however, retrogradely labeled neurons originated only from the TRD injection made in the CNIC (Fig. 2D). The distribution of these labeled neurons extends throughout the central wing of the frequency-band lamina (including both CNIC and DCIC; Fig. 1D), but no labeling was observed in the lateral wing. The total number of neurons labeled in the reconstructed contralateral IC was 174. The absence of labeled neurons in the CNIC from the FD-BDA injection located in the LCIC was not because the FD-BDA injection was ineffective. Examination of more peripheral auditory nuclei that project to the IC revealed that there were FD-BDA-labeled neurons in the ventral cochlear nucleus, superior olivary complex, and nucleus of the lateral lemniscus. Examination of the medial geniculate body, the main ascending projection target of the IC, also revealed an axonal plexus of terminal boutons in the medial division (cf. Kudo and Niimi,^1978^,^1980^; for review see Wenstrup,^2005^). These observations verified that the FD-BDA tracer was effective, so the absence of FL-BDA-labeled neurons in the contralateral IC implies a lack of commissural projections originating from this ventolateral region of the LCIC.

The color-coded density maps of retrogradely labeled neurons in the contralateral IC (Fig. 2D,E) demonstrate that the neurons originating from the TRD injection are distributed in a band with a distinct region of high density. This is confirmed by the normal distribution of labeled neurons in the histograms of neuron distribution for the transverse section in both the X and the Y coordinates (Fig. 2F,G). When the same analysis is made for the sagittal and horizontal sections, the area of maximal neuronal density is located at a point almost symmetrical to the site of the TRD injection in the other IC. Results similar to those from the injection of FD-BDA in case 74 were observed in case 172 (Table 1; data not shown). As for case 74, the injection into the LCIC in case 172 produced no labeled neurons in the contralateral IC.

In case 279 (Fig. 3), we made a TRD injection into the 10.5-kHz lamina of the CNIC and a FD-BDA injection into the equivalent frequency region of the DCIC; both injections resulted in retrogradely labeled neurons in the contralateral IC (Fig. 3A). The number of neurons labeled was 194 for the FD-BDA injection in the dorsal cortex and 186 for TRD injection in the central nucleus, suggesting that the efficacy of the two injections was comparable. However, the neurons labeled by the two tracers were distributed differently in the contralateral IC (Fig. 3A). The FD-BDA-labeled neurons (arising from the DCIC injection) were largely confined to the dorsal cortex and the dorsal most region of the CNIC adjacent to the DCIC, with four or five neurons also labeled in the dorsolateral corner of lateral cortex. In contrast, TRD-labeled neurons (arising from the CNIC injection) extended over the whole lamina and partially overlapped the FD-BDA labeling in the dorsal cortex. The extent of this overlap is clear in these 3D views of the IC reconstructed from all the individual sections in both the transverse and the horizontal projections (Fig. 3A). These data suggest that there are two distinct populations of DCIC neurons in terms of their projections. One group projects almost exclusively to the contralateral DCIC, whereas the other projects to the contralateral CNIC.

Density maps (Fig. 4) show the distribution of labeled neurons from the two injections for case 279. The neurons labeled after the TRD injection into the CNIC give rise to a band of labeled neurons similar to that observed in case 74 (compare the transverse projections from Figs. 2D, 4A). The density of labeling from the DCIC injection (yellow points in Fig. 3A) is significantly reduced toward the central nucleus (Fig. 4B). The location of the maximal density of retrogradely labeled neurons matches the location of the corresponding injection into the contralateral IC (Fig. 3A) in the transverse, horizontal, and sagittal projections. As in case 74, there is an approximately normal distribution of labeled neurons (Fig. 4C,D). This is particularly evident for the TDR neurons. Statistical analysis in the x and y axes confirms that the red and yellow neurons have significantly different distributions (Fig. 4C,D). Inspection of cases 105, 110, and 244 (Table 1), which had similar injections into the CNIC, showed labeling consistent with that of case 279. Of particular interest is case 187 (Table 1; data not shown), in that it had two injections into adjacent, but separate, laminae. The injection in the higher frequency-band lamina was located in the deepest part of the ventral CNIC, and, as in the previous cases, the labeled neurons were distributed almost symmetrically to the injection site, and no labeling was observed in the lateral wing.

Cases 78 and 35 are comparable to one another in that each has two injections in the low-frequency region of the IC. They differ in their location within the IC, but the BF recorded at the injection sites was similar: case 78 had injections in the 2-kHz lamina (Fig. 3B), and case 35 had the injections in the 1.7- and 3-kHz regions (Fig. 3C).

Although the FD-BDA injection in case 78 is slightly larger than the TRD, the former produced more labeled neurons (TRD = 482 neurons vs. FD-BDA = 56). This difference may be explained by the different location of the injections. While the FD-BDA was placed in rostral cortex (RCIC), the TRD injection was sited at the dorsolateral corner of the IC, in the lateral cortex (Fig. 3B). In this case, therefore, both injections were outside the CNIC. Similar to the FD-BDA injection in case 279, the labeling resulting from the TRD injection was widespread and located along the rostrocaudal and mediolateral axes in the dorsolateral corner of the DCIC and LCIC. It forms a cap that leaves the CNIC virtually free of labeling as shown by the 3D reconstruction and the density maps (Fig. 5A). In contrast, the labeling from the FD-BDA injection in case 78 is sparse, but nevertheless it tends to be segregated from that of the TDR injection as shown by the distribution histograms of the labeled neurons (Fig. 5C,D). Statistical comparison of the location of the red and yellow neurons demonstrates that there was a significant difference in their segregation, although it should be noted that the sample size FD-BDA-labeled neurons is small. Sparse labeling in the contralateral IC, similar to that given by the FD-BDA injection into the RCIC in this case (78), was also observed in case 218 (Table 1)

In case 35, the FD-BDA injection was made at BF 2 kHz in the dorsocaudal region near the DCIC-LCIC border, but outside the CNIC. The TRD injection (which was larger than the FD-BDA injection) was made in the equivalent-frequency region of the dorsal cortex (Fig. 3C). The number of retrogradely labeled neurons from the injections in case 35 was 318 for TRD and 32 for FD-BDA. Labeled neurons from both tracers were mostly distributed within the DCIC and, as in case 78, left the CNIC virtually free of labeled neurons, except for a few around its borders. Although there are many more TRD- than FD-BDA-labeled neurons, their positions overlapped extensively. The extent of this overlap becomes clear in the 3D views of the IC reconstructed from all the individual sections. The density maps reveal that there was no single distinct region of maximal labeling (Fig. 6), as does statistical analysis of the histograms along some dimensions. However, the small number of FD-BDA-labeled neurons limits the reliability of these tests.

## DISCUSSION

**Figure 7:**
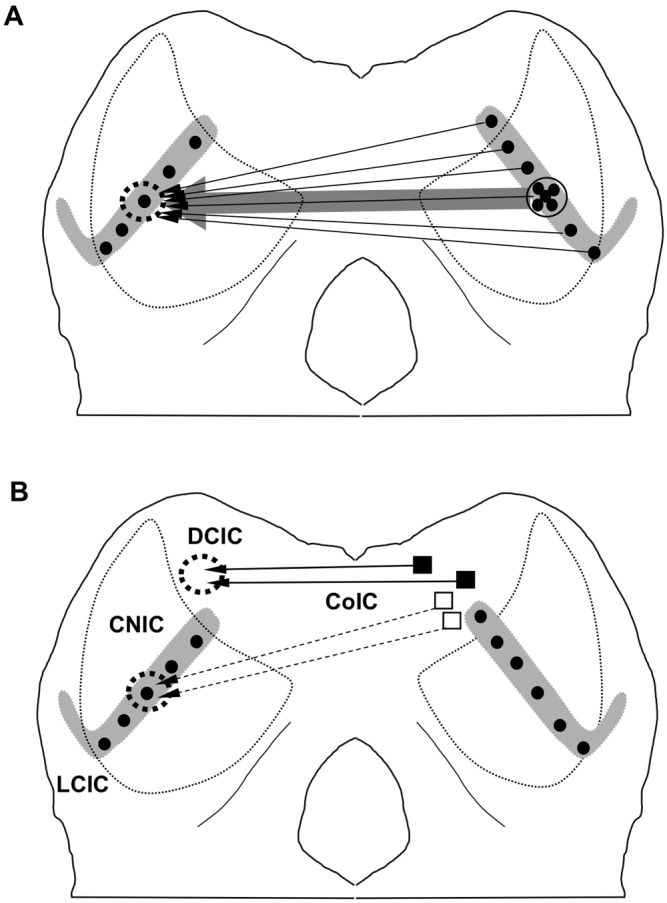
Schematic wiring diagrams of the commissural connections summarizing the main findings of the study. **A**: In the CNIC, the retrograde labeling of neurons demonstrates that an injection into one point on the lamina (dotted circle, left IC) retrogradely labels neurons over the whole extent of the contralateral lamina, consistent with a divergent pattern of connections (thin arrows). Anterograde labeling also supports a divergent projection, insofar as a point injection into the CNIC results in a V-shaped axonal plexus that covers most of the CNIC lamina and extends into the cortices as previously shown (gray free-form shape; Malmierca et al.,1995a; Saldaña and Merchán,^1992^). However, the density of the projection is centered on a point matching the position of the tracer injection. This result is consistent with a point-to-point-weighted wiring pattern (thick arrow). The coexistence of divergent and point-to-point projections suggests two functionally different systems of commissural connections, but from current evidence it is not possible to tell whether they are mediated by a single population or different cell populations. **B**: Our results suggest that two populations of neurons in the DCIC could contribute to the commissural projection to the contralateral IC: one population projects diffusely to the DCIC (solid squares), whereas the other projects to the frequency-band laminae in the CNIC in a tonotopic manner (open squares).

By combining tract-tracing and 3D reconstructions of the locations of retrogradely labeled neurons, we have provided anatomical evidence for the topography of the commissural neurons interconnecting the ICs in rat. Our results suggest that 1) a focal injection into the CNIC labels neurons throughout the equivalent frequency-band laminae in the central nucleus, and in the dorsal cortex on the opposite side, but that 2) the density of the labeled commissurally projecting neurons is weighted toward a point that matches the position of the tracer injection. 3). The results are consistent with there being two populations of commissural neurons in the DCIC (Fig. 7B); one that projects to the frequency-band laminae in the opposite CNIC, with another projecting only to the DCIC. The latter shows a diffuse projection within the DCIC in contrast to the tonotopic organization of the former. 4) Neurons in the LCIC and RCIC make few commissural connections.

The combination of tract-tracing with 3D reconstruction has enabled us to reveal aspects of the commissural connections that were hitherto unknown. Our injections are smaller than those used in previous studies (Adams,^1979^,^1980^; Beyerl,^1978^; Brunso-Bechtold et al.,^1981^; Coleman and Clerici,^1987^; Gonzalez-Hernandez et al.,^1986^) and permitted use of two tracers to study the differential patterns of labeling within different subdivisions in the contralateral IC as demonstrated in other studies of different central nervous system regions (Lanciego and Wouterlood,^2000^,^2006^; Lanciego et al.,1998a,b,^2000^; Schofield et al.,^2007^). TRD and FD-BDA are known to be bidirectional tracers, but both are transported more efficiently in the anterograde direction. In the current study, we take advantage of the often neglected retrograde labeling obtained with these tracers. This less efficient retrograde transport might have had the added advantage of limiting the effective diffusion of the tracer around the injection site (Lanciego and Wouterlood,^2006^; Schofield et al.,^2007^). The use of density maps to represent neuronal distributions, as we have employed here, has previously proved valuable for disclosing details of the organization in other brain areas, including auditory nuclei (for review see Bjaalie et al.,^2006^; Bjaalie and Leergaard,^2006^). Methodological limitations might have affected the total number of neurons labeled: these include the amount of tracer taken up by the neurons from the different injections, the effective size of each injection site, and the possibility that we have missed some double labeling. In the latter case, we can be confident that neurons labeled red have taken up only TRD, but we cannot exclude the possibility that some of the neurons labeled black have taken up TRD as well as FD-BDA. In the current study, density maps enable us to demonstrate not only the spatial extent of the connections but also some quantitative information about the distribution of neurons within those limits.

Previous studies of the CoIC described the overall distribution of commissural neurons but not the fine grain pattern of their projections. Aitkin and Phillips (1984) retrogradely filled commissurally projecting neurons by infiltrating the CoIC with HRP. This study showed filled neurons in all subdivisions of the IC, with a gradient of filled neurons running from ventral to dorsal on moving caudal to rostral in the IC. However, such labeling is not definitive evidence that these neurons project to the other IC, insofar as fibers traveling in the commissure may project to the contralateral auditory thalamus (Kudo and Niimi,^1978^; Oliver,^1984^) as well as to the IC. Coleman and Clerici (1987) reported labeled cells in the DCIC, LCIC, and CNIC following an injection in the contralateral LCIC; however, their injection was very large and located dorsal and rostral in the LCIC, close to the border with the DCIC, so it is possible that the injection encroached on the DCIC and the CNIC. In our case 74, FD-BDA injection was positioned more ventrolaterally in the IC, placing it unambiguously in the LCIC, as confirmed by the expected labeling of terminal axons in the medial division of the auditory thalamus (Kudo and Niimi,^1978^; Oliver,^1984^).

The retrograde labeling of neurons throughout a frequency-band lamina following an injection at a point in the CNIC shows that neurons over the whole plane of the lamina project to each point within the lamina and is consistent with commissural neurons in the CNIC making a divergent pattern of connections to the equivalent contralateral laminae (Fig. 7A, thin arrows). An alternative explanation for this finding is that our injection labels neurons along the whole lamina as a consequence of damage to axons of passage travelling from the commissure to neurons located more laterally in the lamina than the injection site. Although we cannot exclude this interpretation, it seems less likely because in that case one would expect a larger number of stained neurons lateral than medial to the injection site, but this does not appear to be the case. Furthermore, our anterograde labeling also supports a divergent projection, insofar as a point injection into the CNIC results in a V-shaped axonal plexus that covers most of the lamina in the CNIC and extends into the cortices as previously described (Malmierca et al.,1995a; Saldaña and Merchán,^1992^). Imposed upon this underlying divergent pattern of commissural connections, however, there is a greater density of labeled neurons centered on and around the area homotopic to the injection site. Thus, although connections are not exclusively limited to corresponding points on the laminae, corresponding points are represented by more strongly focused connections consistent with a point-weighted pattern (Fig. 7A, thick arrow). It is noteworthy that a pattern similar to the one we describe here is observable in the study of corticocollicular projections in the gerbil by Bajo and Moore (2005). These authors made injections of BDA into the CNIC to retrogradely label the corticofugal projection to the IC. Although the study focused on the corticocollicular projections and did not report the commissurally labeled neurons, detailed inspection of their Figure 6 shows that neurons in the IC contralateral to the injection site are mostly concentrated in a region symmetrical to the injection site, as we have demonstrated here.

It is not possible to tell from the current data whether there are two types of commissural neurons in the CNIC, one type that is solely responsible for the focal, point-to-point projection (Fig. 7A, thick arrow and neurons encircled in the left IC) and another that accounts for the divergent projection (Fig. 7A, thin arrows), or in contrast whether a single neuronal population contributes both projections. Insofar as the distribution is continuous, the former is perhaps more likely.

The coexistence of divergent and point-to-point projections suggests that two functional systems operate across the commissure. One would allow corresponding points on the lamina to interact, whereas the other, more diffuse network would facilitate interactions between neurons over the plane of the lamina. If the IC plays a role in integrating inputs from the lower brainstem, one might expect to find connections that provide the means whereby different input sources can interact.

Our results are therefore consistent with the possibility that, whereas commissural projections emphasize connections between corresponding points on a lamina, they also allow for the integration of information both within a lamina and between the DCIC and the CNIC. An important challenge in understanding the organization of the IC is discovering what is represented over the surface of a frequency-band lamina, i.e., in the plane orthogonal to the tonotopic axis. Evidence exists for a shallow gradient in best frequency across the laminae (Langner and Schreiner,^1988^; Malmierca et al.,^2008^; Merzenich and Reid,^1974^), and other stimulus features have also been reported to be mapped over their surface, including modulation frequency periodotopy (Langner and Schreiner,^1988^; Schreiner and Langner,^1988^), selectivity for rate of change of frequency (Ehret et al.,^2003^; Hage and Ehret,^2003^), and unit threshold and bandwidth of frequency tuning (Schreiner and Langner,^1988^; for reviews see Ehret and Schreiner,^2005^; Rees and Langner,^2005^). The divergent pattern of connections we have observed could be a means by which the integration of responses across such parameter representations is affected.

A second important feature of our results is the differential labeling following injections made in the dorsal cortex and the central nucleus where they occur in a single lamina. A point injection in the central nucleus labels neurons in a laminar pattern in the opposite colliculus. This extends throughout the central nucleus and into the deep dorsal cortex, with only a few labeled cells in the LCIC. In contrast, an injection into dorsal cortex in the same lamina labeled cells predominantly in the dorsal cortex. An alternative explanation for these results is that the commissural connections between the central nuclei and the dorsal cortices are distinct but that the TRD injection into the central nucleus diffused into the dorsal cortex, resulting in labeling in both divisions. However, if this were so, one would expect a similar dispersion of tracer to occur with the FD-BDA injection in the dorsal cortex injection, leading to labeling in the central nucleus, but this is not the case. Furthermore, it is important to emphasize that our injections are smaller than those in previous studies in which larger injections were used to facilitate as much labeling as possible. Such caveats notwithstanding, these results suggest the possibility that there are separate zones within the IC with respect to the distribution of commissural connections. Recent studies by Cant and Benson (2007, 2008) have demonstrated that there are separate zones in the CNIC that differ with respect to their afferent inputs from the auditory brainstem nuclei and their projections to the auditory thalamus, so it is perhaps not surprising that there would be separate zones in the CNIC with respect to the commissural connections as well. This notion is also compatible with the concept of synaptic domains put forward by Oliver and colleagues (Loftus et al.,^2004^; Oliver,^2005^).

The different patterns of labeling observed in the DCIC following injections into the DCIC or the CNIC support the possibility that the DCIC contains two distinct populations of neurons with respect to their commissural connections. One population makes connections along the length of the lamina extending from the DCIC into the CNIC, whereas the other appears to send connections only to the DCIC on the opposite side in a more diffuse manner (Fig. 7B). Definitive evidence for such a pattern of connections, however, would require filling the whole CNIC or the relevant lamina with tracer. Functionally, the DCIC neurons that project to the CNIC could exert an influence over neurons in the CNIC in a frequency-specific fashion, although the second, DCIC-restricted population could facilitate interactions between the two dorsal cortices. Although anatomical studies have recognized three subdivisions of the IC, the frequency-band laminae extend across the borders of these subdivisions and into at least the deep layers of both the dorsal and the lateral cortices (Coote and Rees,^2008^; Malmierca et al.,1995a; Saldaña and Merchán,^1992^). Similarly, physiological studies have shown that the tonotopic organization is not bounded by the borders of the subdivisions (Merzenich and Reid,^1974^). The pattern of the commissural connections that we describe is consistent with this organization, with one population of neurons in the DCIC following the course of the frequency-band lamina, whereas the other may exert a more frequency-independent influence over the frequency-band laminae where they extend into the deeper layers of the DCIC. The influence of the DCIC over the CNIC may be a means whereby corticofugal connections from the auditory cortex (which target predominantly the DCIC; Saldaña et al.,^1996^; Winer et al.,^1998^) exert an influence in the central nucleus on the contralateral side.
